# Management of Enteritis Associated With Tricohepatoenteric Syndrome due to *SKIV2L* Mutation Using the Combination of JAK1/2 Inhibition and Azathioprine

**DOI:** 10.1097/PG9.0000000000000264

**Published:** 2022-10-20

**Authors:** Jonathan Talbot, Anthony E. Wiskin, Marie Monaghan, Anu Goenka, Gillian Rice, Marion Roderick

**Affiliations:** From the * Paediatric Immunology and Infectious Diseases, Bristol Royal Hospital for Children, UK; † Paediatric Gastroentology, Bristol Royal Hospital for Children, UK; ‡ Cellular and Molecular Medicine, University of Bristol, UK; § Department of genetic Medicine, University of Manchester, UK.

**Keywords:** immunology, genetics, tricohepatoenteric syndrome, type 1 interferonopathy

## Abstract

Tricohepatoenteric syndrome is a rare genetic disorder caused by mutations in *SKIV2L* or *TTC37*. An upregulation of type 1 interferon signaling is associated with the *SKIV2L* variation. Introduction of Baricitinib as a JAK1/ 2 kinase inhibitor alongside traditional immunosuppressive agents successfully reduced the symptoms of enteritis by blocking the inflammogenic effects of type 1 interferonopathy in a case of tricohepatoenteric syndrome diagnosed in a 5-year-old boy.

Trichohepatoenteric syndrome (THES) or “syndromic/phenotypic diarrhea” is a rare genetic disorder of autosomal recessive inheritance with an incidence of 1/1 100 000 and approximately 100 cases described in the literature. *SKIV2L* is the second identified gene causing THES alongside *TTC37* (1). The disease is characterized by brittle, woolly hair, liver dysfunction, and intractable watery diarrhea. Patients also exhibit distinctive facial features, immunodeficiency due to defective antibody production, skin abnormalities, intellectual disability, and congenital heart defects (1,2). Upregulated type 1 interferon signaling has been reported in THES caused by mutations in *SKIV2L* but not *TTC37* (3). Type 1 interferon plays a key role in the immune response against viral infections, and thus when dysregulated can result in predisposition to infections or excessive inflammation (3). *SKIV2L* encodes helicase SKI2W, a deadbox protein essential for degrading nonfunctioning cytoplasmic mRNA. Biallelic loss-of-function mutations in *SKIV2L* have been proposed to accumulate cytosolic mRNA, leading to enhanced signaling through intracellular antiviral receptors, and unrestrained production of type 1 interferons that drive inflammation via the JAK-STAT pathway (3). A review of 80 patients with THES described largely supportive management, including elemental and parenteral nutrition, and a significant risk of mortality from infectious complications (4). Steroids were used in 20% of cases without clear benefit. A variety of immunosuppressive drugs (azathioprine, ciclosporin, methotrexate, sirolimus, tacrolimus, and tumor necrosis factor inhibition) were used in 16% of cases, sometimes in combination, with no consistent benefits (4).

We describe a 5-year-old boy with THES, born to nonconsanguineous parents. Throughout infancy he suffered from intractable vomiting and diarrhea requiring parenteral nutrition. Endoscopy showed mixed inflammatory infiltrate in the duodenum with villous atrophy, and chronic gastritis and occasional apoptotic bodies. Sigmoidoscopy revealed no visible mural inflammation. Parenteral methylprednisolone led to improvement in his vomiting, but this was not sustained after transitioning to enteral prednisolone. He was ultimately unable to tolerate any enteral feeds. Gastrointestinal symptoms were exacerbated by frequent upper respiratory tract infections. Inflammatory markers were persistently raised during the first 6 months of life with a median C-reactive protein of 19 mg/L (interquartile range 28 mg/L). Immunological studies showed a serum IgG of 4.65 g/L (normal range 3.0–9.0) at 6 months of age but absent antibody responses to tetanus, diphtheria, pneumococcal conjugate, and *Haemophilus influenzae* type b vaccines, despite booster doses. From the age of 16 months, the patient received replacement intravenous immunoglobulin at a dose of 0.16 g/kg/wk every 3 weeks for antibody replacement in view of his absent vaccine responses. Without enteral feeding, his gastrointestinal symptoms ameliorated. By the age of 2 years, he was vomiting a few times daily and remained unable to tolerate any enteral nutrition. Aged 30 months, enteral feeds were reintroduced as small volumes of amino-acid-based formula; but again, had to be stopped due to frequent, loose, bloody stools with associated discomfort. Despite stopping feeds, he continued to pass bloody stool 10 times daily with additional nocturnal stools. Endoscopy showed active inflammation in the sigmoid and descending colon, at which point duodenal biopsies revealed normal villi and no inflammation. No viral, bacterial, or parasitic organisms were identified from stool cultures. His inflammatory colitis did not respond to oral prednisolone.

Whole exome sequencing identified a homozygous missense variant (c.2147T>A/p.(Ile716Asn)) in exon 18 of *SKIV2L*. Both parents were identified as unaffected heterozygous carriers. Interferon stimulated gene expression was assessed as previously described (5), and found to be upregulated on 2 of 3 occasions: scores of 2.7, 1.5, and 10.7 at 6, 21, and 35 months, respectively, where a score >2.5 represents +2 standard deviations of a healthy control cohort. Given accumulating evidence supporting the efficacy of Janus Kinase (JAK) 1/2 inhibitors such as baricitinib in the treatment of type 1 interferonopathies (6,7), our patient was commenced on baricitinib from the age of 35 months at a dose of 2 mg once daily. This dose represents half an adult daily dose for rheumatoid arthritis and was selected based on trial dosing for juvenile idiopathic arthritis (8). Aside from dosing considerations, side effects of this treatment include a risk of infection including herpes simplex virus, tuberculosis, and urinary tract infections as well as hypercholesterolemia. He was screened for tuberculosis and Hepatitis B before starting therapy, with regular full blood counts, renal function, and liver function monitoring performed alongside his TPN investigations. Within 2 weeks of commencing baricitinib, he no longer produced bloody stools. After 8 weeks of treatment, his stool frequency has reduced from 10 bloody stools to 5 nonbloody stools per day with no nocturnal stooling. He successfully started trophic enteral feeds with no vomiting or increase in stool frequency. No side effects have been reported, and his interferon score was reduced. His dose was increased to 2 mg twice daily after 8 months, given that the half-life of baricitinib is 4.4 hours in individuals with a body weight of <20 kg (8). Despite the initial amelioration in his symptoms, baricitinib alone was not associated with sustained remission. Over the subsequent year, he presented with a further flare of colitis requiring cessation of enteral feeds and corticosteroid treatment. He was commenced on 1.5 mg/kg azathioprine alongside baricitinib 2 mg 3 times daily 30 months after its first administration. This combination of medication has prevented any further flares, allowing the patient to tolerate 110 mL amino-acid-based milk 3 times daily, and reducing the parental nutritional requirement to 6 times per week. He continues to show worsening symptomatology during intercurrent illnesses. He presented with high fevers, malaise, and elevated inflammatory markers lasting >2 weeks due to SARS-CoV-2 infection (Delta variant) age 5 years, but with no evidence of seroconversion to the nucleocapsid protein. He was treated with casirivimab 112 mg/imdevimab 112 mg to prevent deterioration, with cessation of fevers within 24 hours of administration.

This case highlights using a JAK1/2 inhibitor to block inflammation possibly related to enhanced type 1 interferon signaling in cases of THES caused by *SKIV2* mutation. Although baricitinib may not prevent the need for concurrent antimetabolite immunosuppressives, its use was associated with an improvement in our patient’s quality of life by reducing enteritis and parental nutrition dependence. We also demonstrate how monoclonal antibody therapy may be useful in similar patients who present with persistent inflammatory responses, as a functional and regulated interferon response is required to successfully control SARS-CoV-2 infection.
